# TRP Channels in Angiogenesis and Other Endothelial Functions

**DOI:** 10.3389/fphys.2018.01731

**Published:** 2018-12-03

**Authors:** Tarik Smani, Luis J. Gómez, Sergio Regodon, Geoffrey E. Woodard, Geraldine Siegfried, Abdel-Majid Khatib, Juan A. Rosado

**Affiliations:** ^1^Department of Medical Physiology and Biophysic, Institute of Biomedicine of Seville, University of Seville, Sevilla, Spain; ^2^CIBERCV, Madrid, Spain; ^3^Department of Animal Medicine, University of Extremadura, Cáceres, Spain; ^4^Department of Surgery, Uniformed Services University of the Health Sciences, Bethesda, MD, United States; ^5^INSERM U1029, University of Bordeaux, Bordeaux, France; ^6^Cell Physiology Research Group, Department of Physiology, University of Extremadura, Cáceres, Spain

**Keywords:** angiogenesis, endothelial cells, VEGF, TRP channels, TRPC, TRPV, TRPM

## Abstract

Angiogenesis is the growth of blood vessels mediated by proliferation, migration, and spatial organization of endothelial cells. This mechanism is regulated by a balance between stimulatory and inhibitory factors. Proangiogenic factors include a variety of VEGF family members, while thrombospondin and endostatin, among others, have been reported as suppressors of angiogenesis. Transient receptor potential (TRP) channels belong to a superfamily of cation-permeable channels that play a relevant role in a number of cellular functions mostly derived from their influence in intracellular Ca^2+^ homeostasis. Endothelial cells express a variety of TRP channels, including members of the TRPC, TRPV, TRPP, TRPA, and TRPM families, which play a relevant role in a number of functions, including endothelium-induced vasodilation, vascular permeability as well as sensing hemodynamic and chemical changes. Furthermore, TRP channels have been reported to play an important role in angiogenesis. This review summarizes the current knowledge and limitations concerning the involvement of particular TRP channels in growth factor-induced angiogenesis.

## The angiogenic process

The endothelium is a monolayer of endothelial cells (ECs) that line the internal surface of the vascular wall. In addition to serve as a barrier between circulation and the vascular smooth muscle cells, the endothelium plays a relevant role sensing hemodynamic and chemical changes in blood, regulating hemostasis and participating in the formation of new blood vessels, a process called angiogenesis. To create new vessels, ECs need to proliferate, to migrate, and to be organized in three dimensions. There are distinct processes of angiogenesis. The most rapid angiogenic mechanism is known as intussusception. Common in vascular remodeling during development, intussusception is the splitting of a preexisting vessel into two new smaller vessels. This occurs by penetration of smooth muscle cells through the endothelial cell layer (Burri et al., 2004). The formation of new vessels in adult during both physiological and pathological angiogenesis was also attributed to circulating bone marrow—derived endothelial precursor cells (EPCs). Although EPCs are mainly found in active sites of angiogenesis following a chemotactic signal (Patenaude et al., 2010), these cells act as collaborator cells in close proximity to the endothelium and are not incorporated into the vessel (Grunewald et al., 2006). Other angiogenic mechanisms occur during sprouting angiogenesis. Indeed, special ECs of a preexisting vessel acquire the capacity to invade the surrounding tissue by forming an angiogenic sprout. The later is composed of leading cells known as tip cells and trailing stalk cells. These cells are required for the orientation and growth toward the source of an angiogenic factor (Gerhardt and Betsholtz, 2005). As soon as two sprouts anastomose, sprouting is accomplished by lumen formation and the initiation of blood circulation (Fantin et al., 2010). The maturation of newly formed sprouts into differentiated blood vessels requires the recruitment of mural cells, the development of the surrounding matrix and specialization of ECs in organ-specific manner. Pericytes participate in the stabilization of the newly formed blood vessels through direct physical contact and paracrine signaling.

Angiogenesis is regulated by a balance between stimulatory and inhibitory factors. When this balance shifts in favor of positive stimuli the “angiogenic switch” occurs (Hickey and Simon, 2006). To date several negative regulators of angiogenesis have been identified, however little is known about their exact role during physiological angiogenesis. Among these regulators, thrombospondin, previously reported to be secreted by epithelial cells, was found to inhibit tumor growth angiogenesis (Henkin and Volpert, 2011). Lately other anti-angiogenesis agents were also identified including endostatin, tumstatin, vasostatin, and lately anti-vascular endothelial growth factor (VEGF) (Norden et al., 2009). In the adult, under physiological conditions blood ECs are quiescent due to the increased levels of anti-angiogenic factors (thrombospondin and endostatin) compared to proangiogenic forces, such as the VEGF-A, placental growth factor (PlGF), platelet-derived growth factor (PDGF), and others. During pathological situations, including carcinogenesis and chronic inflammation, angiogenic factors are upregulated, and become more prominent than anti-angiogenic agents.

## VEGF family members and their receptors

**Figure 1:**
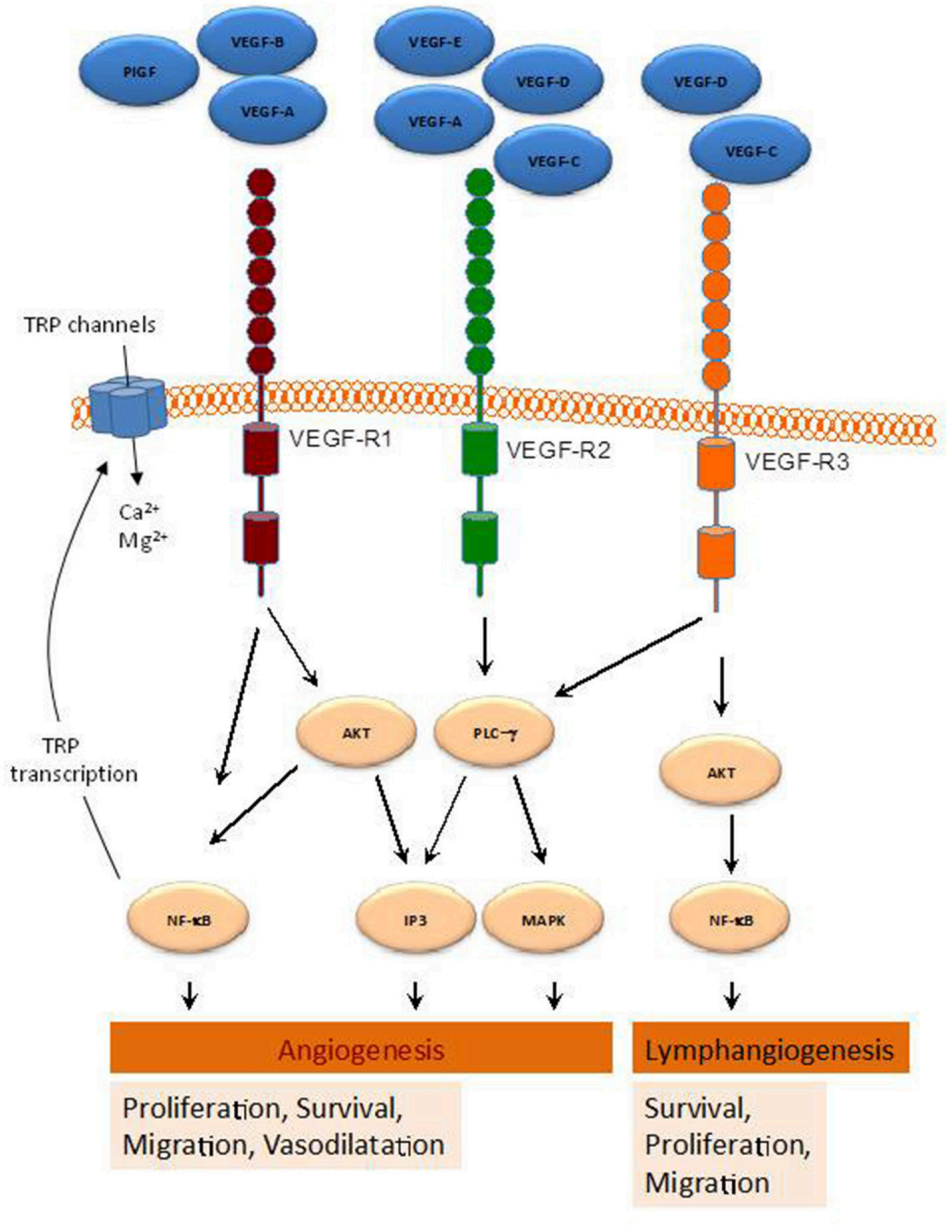
Schematic representation of various pathways activated by the VEGF-family members. By binding to their receptors (VEGFR1-3), indicated VEGF members activate several intracellular pathways involved in a range of cellular functions leading to angiogenesis and lymphangiogenesis.

The growth factors VEGFs, PDGF-BB, and PlGF are all grouped in the VEGF superfamily (McDonald and Hendrickson, 1993), and contain a cystine knot motif in their amino acid sequence. In mammals five VEGF members have been identified, namely VEGF-A, -B, -C, -D, and PlGF (McDonald and Hendrickson, 1993) (Figure 1). These growth factors mediate their function on vascular and lymphatic ECs through their cognate receptors VEGFR-1, -2, and -3 and the NP co-receptors. VEGF-A is able to activate both VEGFR-1 and VEGFR-2, whereas VEGF-B and PlGF are selective ligands for VEGFR-1 (Takahashi and Shibuya, 2005). VEGF-C and -D are the only known ligands for VEGFR-3 and are also able to activate VEGFR-2 (Tammela et al., 2005). The different expression of these receptors in various tissues seemed to be responsible for the relatively specific function of their ligands. Indeed, VEGFR-1 and VEGFR-2 are mainly found in vascular ECs, VEGFR-3 is largely restricted to lymphatic endothelium. Thus, according to their affinities for VEGFR-1 and -2, VEGF-A, -B, and PlGF exert angiogenic activities, while VEGF-C and -D predominantly act as lymphangiogenic growth factors by activating VEGFR-3. The interaction of VEGF with VEGFR (Jakobsson et al., 2006) leads to receptor dimerization leading to conformational changes and phosphorylation of their tyrosine residues, which is important for downstream signal mediators activation. The activation cascades outcome is the elaboration of various VEGF biological responses such as cell proliferation, survival, migration and ECs arrangement to form vascular tubes. The activation of VEGFR can be repressed by its dephosphorylation mediated by various phosphotyrosine phosphatases (PTPs), including density enhanced phosphatase 1 (DEP1) and vascular endothelial PTP (VEPTP) (Kappert et al., 2005).

### VEGFR1

VEGFR1 (also known as Fms-like tyrosine kinase 1, Flt1,) binds VEGF-A, VEGF-B, and PlGF (Wiesmann et al., 1997). Activation of this receptor was found to induce various kinases including phosphoinositide 3′ kinase (PI3K)/protein kinase B (PKB/AKT), extracellular signal-regulated kinase (ERK)/mitogen-activated protein kinase (MAPK), and the stress kinase p38MAPK (Tchaikovski et al., 2008). VEGFR1 exists as a soluble form (sFlt1) (Kendall and Thomas, 1993), that exhibits higher affinity for VEGFA than VEGFR2. As a result, sFlt1 operates as a negative regulator of angiogenesis by reducing VEGFA/VEGFR2 interaction (Ambati et al., 2006).

### VEGFR2

VEGFR2 [KDR (kinase insert domain receptor, human) and Flk1 (fetal liver kinase-1, mouse)]. Actively involved in vascular permeability, this receptor is crucial for ECs function during development. VEGFR2 is expressed most prominently in vascular ECs, with highest expression levels during embryonic vasculogenesis and angiogenesis (Millauer et al., 1993). VEGFR2 expression was also found increased during pathological processes associated with neovascularization such as tumor angiogenesis (Plate et al., 1993). VEGFR2 binds VEGF-A via its extracellular Ig-like domains 2 and 3, but with a lower affinity than VEGFR1 (Fuh et al., 1998). In contrast to VEGFR1, VEGFR2 binds also VEGF-C and VEGF-D (McColl et al., 2003) and represses binding to VEGFR3, which results in the inhibition of the proliferation of lymphatic ECs (Albuquerque et al., 2009). Interaction of VEGF-A and VEGFR2 promotes receptor dimerization (Yang et al., 2010), allowing receptor activation leading to several signaling mediators activation like PLCγ (Cunningham et al., 1997), and the adapter proteins SHB and SCK (Warner et al., 2000). These signals are required for various EC functions including proliferation, cell survival and migration, and vascular permeability.

### VEGFR3

(also known as Flt4) binds VEGF-C and VEGF-D. Produced as precursor proteins, when proteolytically cleaved show increased affinity for both VEGFR2 and VEGFR3 (Joukov et al., 1997). VEGF-C and VEGFR3 interaction is critical for lymphendothelial function. Expressed in vascular ECs VEGFR3 is up-regulated during active angiogenesis. Binding of VEGF-C or VEGF-D to VEGFR3 leads to various kinases activation in VEGFR3 (Dixelius et al., 2003) and the activation of the PI3K/AKT pathway (Mäkinen et al., 2001), critical in lymphendothelial cell migration and sprouting of lymph EPCs and development of the lymphatic system (Karkkainen et al., 2004). Furthermore, VEGF-C-mediated AKT activation is required for embryonic and adult lymphangiogenesis (Zhou et al., 2010).

Compelling evidence demonstrated that VEGFRs increase intracellular Ca^2+^ concentration ([Ca^2+^]_i_), through the activation of TRP and other Ca^2+^ channels, which modulates signaling pathways leading to angiogenesis (Simons et al., 2016). For instance, VEGF-A enhances inositol 1,4,5-trisphosphate (IP_3_) generation, which results in Ca^2+^ store depletion and the activation of store-operated Ca^2+^ entry in ECs and EPCs (SOCE) (Faehling et al., 2002; Moccia et al., 2014a). Consistent with this, SOCE inhibition or removal of extracellular Ca^2+^ has been reported to prevent VEGF-mediated Ca^2+^ oscillations in endothelial colony forming cells (Dragoni et al., 2011). Moreover, TRPC6 has been found to mediate VEGF-induced Ca^2+^ influx in microvessel ECs (Pocock et al., 2004), and both TRPC3 and TRPC6 mediate Ca^2+^ entry by VEGF in human microvascular ECs *in vivo* (Cheng et al., 2006). Furthermore, Mg^2+^ influx through TRP family members, such as TRPM6 and TRPM7, has been provided to be relevant for EC proliferation and angiogenesis (Nilius et al., 2003). TRP channels and VEGF signaling exhibit a cross relationship, so that VEGFRs activation has been reported to induce NFκB-mediated activation of transcription of certain *TRP* genes (Santoni et al., 2011) (Figure 1), while Ca^2+^ influx via TRP channels has been found to stimulate the transcription of genes encoding different growth factors, including VEGF and PDGF, in ECs (Yao and Garland, 2005). Therefore, TRP channels play a relevant role in VEGF-mediated signaling in ECs, as summarized below.

## Overview of the TRP superfamily of cation channels

In 1969, Cosens and Manning reported their findings concerning a blind mutant strain of *Drosophila melanogaster* whose external appearance and histological sections of retinal structure were indistinguishable from the wild-type strain but exhibited abnormal electroretinogram (Cosens and Manning, 1969). Further studies revealed that while short stimuli induce a similar response in the wild-type and mutant fly, the response in the mutant fly to longer light stimulation was characterized by a marked decay in the receptor potential in the presence of illumination. The *trp* mutant, called so due to the transient receptor potential in response to light found in the retinular cells of the mutant strain, as compared to the more sustained receptor potential recorded in the wild-type fly, exhibited a defect in the process that links excitation to the membrane conductance (Minke et al., 1975; Minke, 1977). Later on, the light-sensitive conductance in *Drosophila* photoreceptors was found to be mediated by the Na^+^ and Ca^2+^-permeable channel trp and its homolog trpl (Hardie and Minke, 1992; Phillips et al., 1992), and comprises two distinct currents: one is conducted by the highly Ca^2+^ selective trp channel while the second is conducted by the trpl channel, which is supposed to be responsible for the residual light-sensitive current in the trp mutants (Katz et al., 2017).

The first mammalian homolog of *Drosophila* trp was identified in mouse in 1995 (Petersen et al., 1995) and two independent groups identified the first human transient receptor potential (TRP) channel, called TRPC1, (Wes et al., 1995; Zhu et al., 1995). Since the discovery of the first TRP channel in mammalian cells 28 TRP genes have been identified, which can be grouped into three subfamilies closely related to *Drosophila* trp (TRPC, TRPV, and TRPM), two more distantly related subfamilies (TRPP and TRPML), and a less related TRPN group expressed in flies and worms (Montell et al., 2002; Salido et al., 2011).

**Figure 2:**
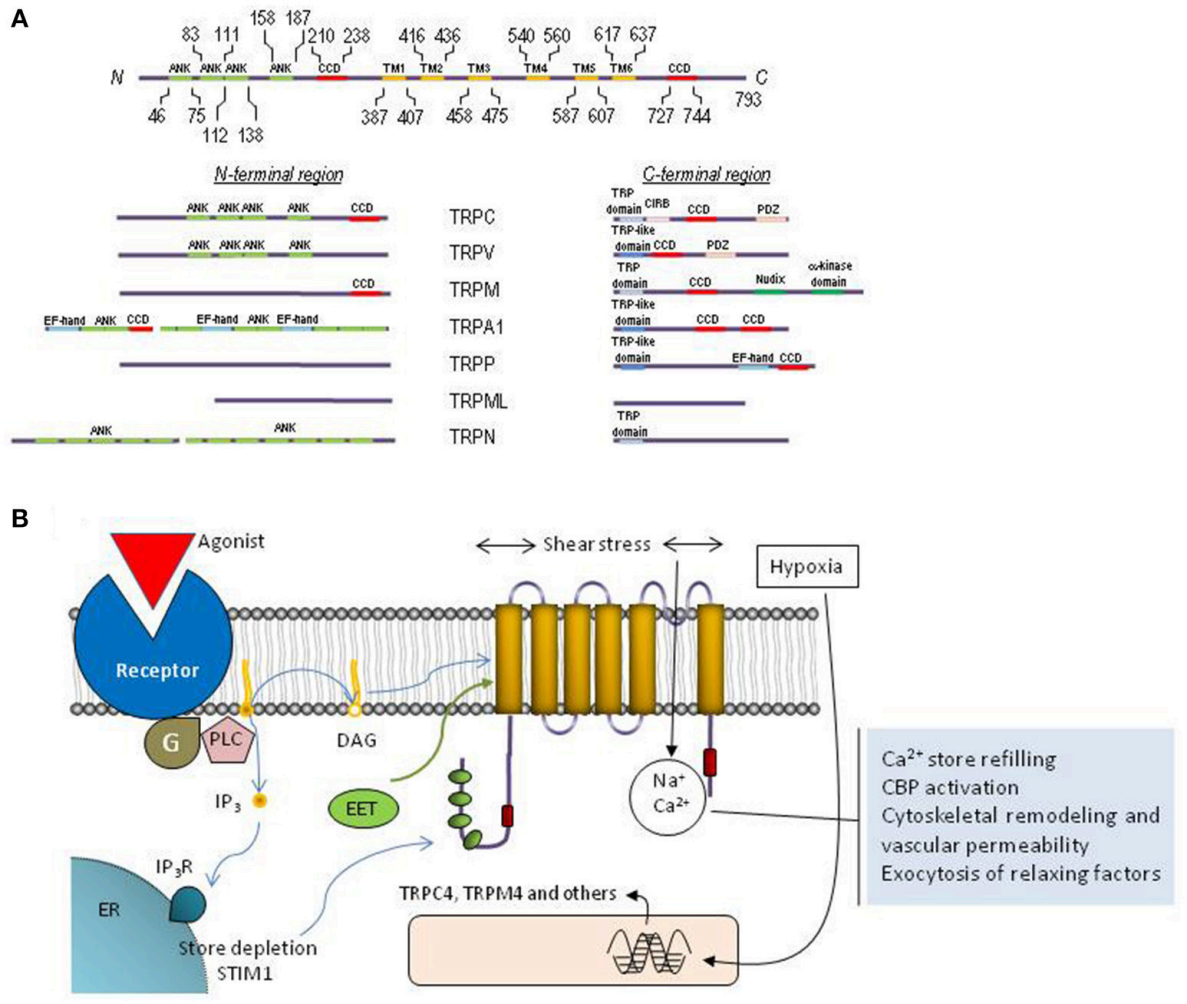
Molecular structure and function of TRP channels. **(A)** Schematic representation of TRPC1 depicting functionally relevant domains. The cytoplasmic N- and C-termini of each TRP family contain different structural and functional domains as indicated. **(B)** Cartoon representing the topology of a TRP monomer within the plasma membrane, activation and functions associated to TRP channels in endothelial cells. The protein exhibits six transmembrane domains (TM1-TM6) with the pore located between transmembrane domains 5 and 6, and both N-terminal and C-terminal domains located in the cytosol. Endothelial TRP channels can be activated by receptor occupation, which, in turn, leads to the activation of phospholipase C and the generation of DAG and inositol 1,4,5-trisphosphate (IP_3_). DAG is an endogenous activator of certain TRP channels and Ca^2+^ store depletion results in STIM1-dependent activation of TRPC1. Furthermore, TRP channels in endothelial cells can be activated by shear stress and the different EETs or their expression can be upregulated by hypoxia. Calcium influx via TRP channels is involved in Ca^2+^ store refilling, activation of Ca^2+^-binding proteins (CBP), cytoskeletal remodeling and the regulation of vascular permeability as well as the exocytosis of smooth muscle cell relaxing factors.

All TRP channels show a common architecture. They are membrane proteins with six putative transmembrane domains (TM1–TM6) and present a cation-permeable pore region created by a loop between TM5 and TM6 (Figure 2). The N- and C-termini are located intracellularly and show a great variability both in length and amino acid sequence among the different TRP members. The N- and C- terminal sequences include a variety of functional domains (Ramsey et al., 2006), including: (1) a variable number of ankyrin repeats (present in the members of TRPA, TRPC, TRPV, and TRPN subfamilies) that have been found to play a relevant role in channel sensing and gating (Gaudet, 2008); (2) TRPC, TRPM and TRPN exhibit a “TRP domain” sequence adjacent to the TM6, which shows highly conserved sequences called TRP boxes 1 and 2, and has been shown to be required for channel tetramerization and function (Venkatachalam and Montell, 2007). Similarly, the TRPV1, TRPA1, and TRPP channels show a TRP-like domain, which shows a similar α-helical configuration and function to TRP domains (García-Sanz et al., 2004; Zheng et al., 2018); (3) an α-kinase domain present in TRPM6 and TRPM7 that regulates channel function and sensitivity to Mg^2+^·ATP (Clark et al., 2008; Zhang et al., 2014); (4) an ADPR hydrolase domain (Nudix-like domain or NUDT9 homology domain) in TRPM2, which has been reported to sense ADP-ribose concentration and convey this information to the cell by activation of cation entry (Scharenberg, 2005); (5) a calmodulin- and IP_3_ receptor (IP_3_R)-binding site (CIRB, present in TRPC, members), a domain that has been reported to be involved in the modulation of TRPC6 channel function by IP_3_R and Ca^2+^/calmodulin (Dionisio et al., 2011) and to modulate plasma membrane location of TRPC3 channels via an IP_3_R-independent pathway (Wedel et al., 2003); (6) an EF-hand Ca^2+^-binding domain (present in members of the TRPP, TRPML, and TRPA1) (Zurborg et al., 2007); (7) a large extracellular loop between TM1 and TM2 in TRPP and TRPML, which has recently been reported to play an essential role in channel assembly and function (Salehi-Najafabadi et al., 2017); and, (8) coiled-coil domains located in the C-terminal region (for TRPV, TRPM, TRPA1, and TRPP) or in the N- and C-terminal domains (for TRPC) (García-Sanz et al., 2004; Li et al., 2011a) (Figure 2), which have been found to be involved in subunit-subunit interaction (Launay et al., 2004), as well as in the interaction of TRPs with channel modulators, such as the interaction of TRPC proteins with the endoplasmic reticulum Ca^2+^ sensor, STIM1 (Lee et al., 2014).

Mammalian TRP channels are permeable to monovalent and divalent cations, with a permeability for Ca^2+^ over Na^+^ (ratio P_Ca_/P_Na_) that ranges from channels that are selective for monovalent cations, such as TRPM4 and TRPM5, to highly Ca^2+^ selective channels, including TRPV5 and TRPV6, which exhibit a ratio P_Ca_/P_Na_ over 100 (Freichel et al., 2012). It has also been reported that TRP channels are permeable to metal ions, such as manganese, magnesium, zinc, barium, strontium, nickel or cobalt, and, certain TRP members exhibit a greater relative permeability for these ions than for Ca^2+^ (for an extensive review see Bouron et al., 2015).

TRP channels have been reported to be activated and/or modulated by a number of chemical and physical stimuli, such as extracellular and intracellular ions (including H^+^, Ca^2+^ and Mg^2+^) (Liman, 2007; Zhang et al., 2014) and ligands, both intracellular molecules [such as diacylglycerol (Hofmann et al., 1999), phosphoinositide-4,5-bisphosphate (PIP_2_) (Nilius et al., 2006; Jardín et al., 2008a)] and exogenous natural and synthetic ligands (for a review see Harteneck et al., 2011; Vetter and Lewis, 2011), temperature and mechanical stretch (Venkatachalam and Montell, 2007). Furthermore, TRPC channels have been reported to be activated by intracellular Ca^2+^ store depletion via the interaction with STIM1 and Orai1, the key elements for the activation of store-operated Ca^2+^ entry (SOCE) (Zhang et al., 2005; Feske et al., 2006). Ca^2+^ entry through SOCE is conducted by two types of channels: the highly Ca^2+^ selective CRAC (Ca^2+^ release-activated Ca^2+^) channel, involving Orai1 subunits, and the less selective store-operated Ca^2+^ (SOC) channels (Desai et al., 2015). Despite the participation of TRPC channels in SOCE has been a matter of intense debate in the past, there is now a general consensus that TRPC1 is a component of the SOC channels, forming a ternary complex with Orai1 and STIM1, which confers store depletion sensitivity to SOC channels (Huang et al., 2006; Jardin et al., 2008b; Desai et al., 2015; Ambudkar et al., 2017).

## TRP channels in the endothelium

ECs have been reported to express at the transcript and/or protein level most of the mammalian TRP isoforms identified, including TRPC1, 3, 4, 5, 6, and 7, TRPV1, 2, and 4, TRPP1 and 2, TRPA1 and TRPM1, 2, 3, 4, 6, 7, and 8, although differences in the expression profile have been reported for different vasculatures and species (Wong and Yao, 2011; Cao et al., 2018).

TRP channels contribute to the Ca^2+^ influx induced by a plethora of vasoactive agents, including thrombin, ATP, angiotensin II or bradykinin (Bishara and Ding, 2010; Sundivakkam et al., 2013). Ca^2+^ entry through TRP channels has been found to be involved in the activation of a number of signaling pathways and cellular functions. Among the major functional roles of ECs is the modulation of the vascular tone through the release of a variety of factors that induce relaxation of smooth muscle cells. TRP channels have been reported to play an important role in this process, for instance, irisin, an exercise-induced myokine, has been reported to induce vasodilatation of rat mesenteric arteries through the activation of endothelial TRPV4 channels, which are involved in Ca^2+^ influx induced by irisin in primary cultured rat mesenteric artery ECs (Ye et al., 2018). Furthermore, TRPV4-deficient mice exhibit attenuated acetylcholine-induced endothelium-dependent vasodilatation associated to a reduced nitric oxide (NO) release (Zhang et al., 2009).

TRP channels have also been found to play a relevant role in vascular permeability, a cellular process that is based on transcellular and paracellular pathways, being the later regulated by the balance between cell-cell adhesive forces and contractile forces generated by the endothelial cytoskeleton (Wong and Yao, 2011). Probably, one of the most widely investigated TRP channels for its implication in endothelial permeability is TRPC6, which has been shown to be involved in lung ischemia-reperfusion-induced edema in mice (Weissmann et al., 2012), as well as in endotoxin-induced lung vascular permeability (Tauseef et al., 2012). TRPC1 and TRPC4 have also been found to be involved in vascular permeability. Expression of TRPC1 induced by TNFα has been reported to enhanced Ca^2+^ influx and vascular permeability (Paria et al., 2003) and TRPC4-deficient mice where thrombin-evoked Ca^2+^ signals and endothelial permeability were reduced (Tiruppathi et al., 2002). Other TRP channels, such as TRPV4 or TRPM4, have been reported to play a relevant role in vascular permeability. In isolated rat lung, activation of TRPV4 by 4α-phorbol 12,13-didecanoate (4α-PDD), as well as by 5,6- or 14,15-epoxyeicosatrienoic acids, has been found to increase lung endothelial permeability in a Ca^2+^ entry-dependent manner, which indicates that TRPV4 is involved in the disruption of the alveolar septal barrier. Consistent with this, the effect of the TRPV4 agonists was impaired in TRPV4-deficient mice (Alvarez et al., 2006). TRPM4 has been reported to be up-regulated in the ECs of blood vessels following spinal cord injury, which has been associated to secondary hemorrhage and progressive hemorrhagic necrosis (Gerzanich et al., 2009). Although the mechanism underlying the role of TRPM4 in vascular permeability remains unclear, there is a body of evidence supporting that TRPM4 expression is involved in post-trauma secondary hemorrhage, i.e., after spinal cord injury in rats, *in vivo* gene suppression using Trpm4 antisense was found to preserve capillary integrity and impair secondary hemorrhage, and similar results were observed in TRPM4-deficient mice (Gerzanich et al., 2009). Furthermore, 17β-estradiol, which attenuates TRPM4 and sulfonylurea receptor-1, has been reported to suppress disruption of the blood-spinal cord barrier and attenuate secondary hemorrhage after spinal cord injury (Lee et al., 2015).

Finally, TRP channels have also been reported to play a functional role in the ability of the ECs to sense hemodynamic and chemical changes. Flow shear force results in rises in cytosolic Ca^2+^ concentration ([Ca^2+^]_i_), which, in turn, lead to the release of vasodilating factors. A number of TRP channels are sensitive to flow shear stress, such as TRPV4. It has been reported that flow shear stress induces relaxation of the carotid artery, an effect that is mimicked by the TRPV4 activator 4α-PDD and is prevented by the non-selective TRPV4 inhibitor ruthenium red (Köhler et al., 2006). The involvement of TRPV4 in endothelial-dependent vascular dilation was confirmed in TRPV4-deficient mice, which exhibit attenuated response to stimulation with endothelium-derived hyperpolarizing factor (Loot et al., 2008) and, more recently, with studies reporting that TRPV4-TRPC1 heteromeric channels mediate flow shear-induced endothelial Ca^2+^ influx by a mechanism that might involve an upstream mechanosensitive pathway including phospholipase A2 and cytochrome P450 epoxygenase activity (Loot et al., 2008; Ma et al., 2010). The TRPP1-TRPP2 complex has also been suggested to play a role in flow-induced ECs-mediated vascular dilation, as Ca^2+^ influx and NO production in response to flow is significantly reduced by TRPP1 or TRPP2 expression silencing (Nauli et al., 2008; AbouAlaiwi et al., 2009); although the mechanism underlying the activation of TRPP2-mediated Ca^2+^ entry by flow shear forces in ECs remains unclear. A more recent study has identified the formation of a heteromeric channel including the flow-sensitive TRPV4 and both TRPC1 and TRPP2, which mediates the flow-induced Ca^2+^ influx in native vascular ECs (Du et al., 2014). TRP channels also play a relevant role sensing chemical blood components. For instance, TRPC3, TRPC4, TRPM2, TRPM7, and TRPA1 have been reported to be activated by oxidative stress, leading to Na^+^ and Ca^2+^ entry and, thus, mediating the vascular effects associated to reactive oxygen species (ROS) (Wong and Yao, 2011). On the other hand, in addition to sensing ROS, TRPA1 channels have been found to detect molecular oxygen and are essential for hyperoxia- and hypoxia-induced vagal responses (Takahashi et al., 2011). The mechanistic details of the activation of TRPA1 by O_2_ as well as the transduction pathway remain unclear; however, in cerebral arteries, TRPA1 in the endothelium is mostly located within myoendothelial junction sites, where TRPA1-mediated Ca^2+^ influx is associated to endothelium-dependent smooth muscle cell vasodilatation through the activation of Ca^2+^-activated K^+^ channels (K_Ca_3.1), which, in turns, results in ECs hyperpolarization that is conducted via myoendothelial gap junctions to hyperpolarize the adjacent smooth muscle cell, resulting in myocyte relaxation (Earley, 2012).

## TRP channels in angiogenesis

TRP channels have also been found to play a relevant role in angiogenesis. Compelling evidence demonstrated that angiogenic growth factors activate TRP channels, causing a subsequent rise in endothelial [Ca^2+^]_i_, which modulates the signal transduction pathways leading to angiogenesis (Kwan et al., 2007). It is known that both tumor and physiological angiogenesis are initiated in hypoxic environment principally due to secretion of several growth factors, such as VEGF. These growth factors stimulate proliferation, migration, and tube formation of ECs, resulting in the generation of new capillary (Kohn et al., 1995). Most studies used particularly VEGF to investigate neovascularization in different experimental model. Briefly, tyrosine phosphorylation of VEGFR triggers activation of phospholipase C (PLC), inositol 1,4,5-triphosphate (InsP3) and diacylglycerol (DAG) generation. The consequent Ca^2+^ entry following the classic Ca^2+^ release modulates signaling pathways leading to angiogenesis (Simons et al., 2016). Several reports demonstrated that VEGF-induced Ca^2+^ entry through different isoforms of TRP in several cell types, such as TRPC3 and TRPC6 (Hamdollah Zadeh et al., 2008; Andrikopoulos et al., 2017); TRPM2 through reactive oxygen generation (Mittal et al., 2015); or TRPV1 (Garreis et al., 2016). Certainly, in ECs some TRPs associate to others isoforms forming heteromeric channels (Loot et al., 2008; Nauli et al., 2008; AbouAlaiwi et al., 2009; Ma et al., 2010), however most studies of angiogenesis focused on only one isoform of TRPs as detailed below.

### Role of TRPCs

The participation of TRPC3 in angiogenesis has been characterized in Human Umbilical Vein ECs (HUVEC) treated with VEGF. TRPC3 inhibition or its silencing with siRNA attenuated VEGF activation of ERK1/2 phosphorylation, and stimulation of [Ca^2+^]_i_ transients in HUVEC. Additionally, siRNA of TRPC3 significantly suppressed endothelial tube formation, an indicator of angiogenesis (Andrikopoulos et al., 2017). This study suggests that TRPC3 is activated by the generation of DAG downstream of VEGFR in HUVECs, causing Na^+^ influx by subsequent activation of the Na^+^/Ca^2+^ exchanger in reversal mode, contributing ultimately to angiogenesis (Andrikopoulos et al., 2017). The role of TRPC3 in angiogenesis has also been evaluated in EPCs (Dragoni et al., 2013). As stated above, EPCs are adult stem cells having the ability to differentiate into ECs, and thereby they promote postnatal vasculogenesis and endothelial repair after vascular intima injury (Djohan et al., 2018). Molecular and pharmacological inhibition of TRPC3, using siRNA and Pyr3 respectively, abrogated VEGF-induced Ca^2+^ response and inhibited proliferation of EPCs (Dragoni et al., 2013). The selectivity of Pyr3 on TRPC3 might be questioned, nevertheless the effect of TRPC3 silencing suggest that this channels might be relevant for vasculogenesis.

Independently of ECs stimulation with VEGF, silencing the expression of TRPC3, TRPC4, or TRPC5 also prevented spontaneous [Ca^2+^]_i_ oscillations and inhibited tube formation in human umbilical vein-derived EC line EA.hy926 and HUVECs (Antigny et al., 2012). A recent study performed in retina microvascular ECs showed that hypoxia, a potent trigger of angiogenesis, enhanced the expression of TRPC4, whose silencing inhibited VEGF-induced ECs proliferation and migration and *in vitro* angiogenesis evaluated by tube formation (Song et al., 2015). More recently, silencing of TRPC4 attenuated oxLDL-induced human coronary ECs proliferation; migration and *in vit*ro angiogenesis-tube formation on matrigel, suggesting that suppression of TRPC4 might be an alternative therapeutic strategy for atherosclerotic neovascularization (Qin et al., 2016).

TRPC6 seems also critical for angiogenesis and Ca^2+^ entry in response to VEGF and 1-oleoyl-2-acetyl-sn-glycerol (OAG, a membrane-permeant DAG analog) in human microvascular ECs and in HUVEC. Experiments using a dominant-negative mutant of TRPC6, made with three mutations in the pore region, reduced ECs proliferation, migration and sprouting in matrigel assay (Hamdollah Zadeh et al., 2008). Similar results were observed in HUVEC, where a dominant-negative form of TRPC6 inhibited VEGF-induced cation current, HUVEC growth and proliferation, as well as VEGF-evoked capillary formation *in vitro* (Ge et al., 2009). The role of TRPC6 in ECs proliferation and tube formation was also observed when 11,12-EET (11,12-cis-epoxyeicosatrienoic acid) was used to stimulate ECs (Ding et al., 2014).

Other studies have focused on the role of TRPC1 in angiogenesis. Indeed, a proangiogenic role for TRPC1 has been described *in vivo* in zebrafish, where authors have identified severe angiogenic defects in intersegmental vessel sprouting after knockdown of TRPC1 (Yu et al., 2010). Furthermore, TRPC1 likely controls cell proliferation and tubulogenesis in normal EPCs and in those isolated from peripheral blood of tumor patients (Moccia et al., 2014b). Recently, *in vivo* matrigel assay confirmed that EPCs isolated from TRPC1 knockout mice has substantially reduced functional activities, including migration and tube formation, indicating that TRPC1 plays an important role in angiogenesis (Du et al., 2018). Nevertheless, other studies suggested that TRPC1 is not relevant for angiogenesis. The use of siRNAs, dominant-negative mutants or neutralizing antibodies, failed to demonstrate that TRPC1 is required for VEGF-induced Ca^2+^ increase in HUVECs and tube formation (Li et al., 2011b; Antigny et al., 2012). Interestingly, TRPC1 knockout mice developed normal vasculature (Schmidt et al., 2010). Therefore, more investigations are still required to clarify the real role of TRPC1 in the angiogenic processes.

### Role of TRPVs

TRPV4 has long been known to regulate angiogenesis and neovascularization by stimulating ECs proliferation and migration as reviewed recently (Moccia, 2018). TRPV4 plays an important role in cytoskeletal reorganization and changes in cell adhesion, which coordinate ECs proliferation and motility via mechanotransduction (Köhler et al., 2006; Reddy et al., 2015; Adapala et al., 2016; Thoppil et al., 2016). TRPV4 is dramatically up-regulated in breast tumor-derived ECs, and is required for arachidonic acid (AA)-evoked Ca^2+^ entry, which increase the rate of ECs migration and motility as compared to control ECs (Fiorio Pla et al., 2012). Moreover, the absence of TRPV4 in knockout mice was associated with an increase in basal Rho/Rho kinase activity, significant increase in ECs proliferation, migration, and abnormal tube formation *in vitro* (Thoppil et al., 2016). Interestingly, another study from the same group confirmed that overexpression or pharmacological activation of TRPV4, using GSK1016790, restored the aberrant ECs mechanosensitivity, migration and normalized tube formation in matrigel assay. TRPV4 activation and overexpression likely normalized the abnormal angiogenesis evoked by tumor ECs through the inhibition of the exacerbated Rho activity (Adapala et al., 2016). Therefore, TRPV4 activation seems relevant to normalize tumor angiogenesis via modulation of Rho/Rho kinase pathway.

TRPV1 has been found to be pro-angiogenic. Intraperitoneal injection of mice with a TRPV1 ligand, evodiamine, promoted vascularization in matrigel plugs used *in vivo* in wild type mice. In contrast, the induced angiogenesis was markedly reduced in TRPV1 knockout mice (Ching et al., 2011). Similarly, using knockout mice TRPV1 appears crucial for 14,15-EET-induced Ca^2+^ influx, NO production and angiogenesis evaluated by tube formation and *in vivo* matrigel assays (Su et al., 2014a). In addition, in human microvascular ECs TRPV1 activation is involved in simvastatin-activated Ca^2+^ influx, which induced the activation of CaMKII signaling and enhanced the formation of TRPV1–eNOS complex, leading to NO production and *in vitro* angiogenesis-tube formation (Su et al., 2014b).

### Role of TRPMs

TRPM2, TRPM4, and TRPM7 have also been found to be involved in angiogenesis (Zhou et al., 2014). Recently, a study demonstrated that VEGF stimulated ECs migration and induced ROS-dependent Ca^2+^ entry through TRPM2 activation. In addition, they showed that matrigel plugs supplemented with VEGF injected subcutaneously in TRPM2 knockout mice presented significantly reduced vessel formation compared to wild type mice. Using the mouse aortic ring assay, they also observed defective capillary sprouting and reduced capillary lengths isolated from TRPM2 knockout mouse rings as compared with WT mice, indicating that TRPM2 was required for angiogenesis and ischemic neovascularization (Mittal et al., 2015). Moreover, TRPM4 is upregulated in vascular endothelium following hypoxia/ischemia *in vitro* and *in vivo*, and in HUVECs following oxygen–glucose deprivation. Pharmacological blocking of TRPM4, or its silencing with siRNA, enhanced tube formation on matrigel and improved capillary integrity *in vivo* (Loh et al., 2014). Previously, a report demonstrated that silencing of TRPM7, mimics the effect of Mg^2+^ deficiency in microvascular ECs growth and migration, proposing magnesium and TRPM7 as a modulator of angiogenesis (Baldoli and Maier, 2012).

### Others TRPs' role in angiogenesis

Little is known regarding the participation of TRPA and TRPP isoforms in the angiogenic process. Few years ago, TRPA1 was suggested as the downstream effector for simvastatin—evoked activation of TRPV1-Ca^2+^ signaling in ECs, since its inhibition markedly decreased eNOS activation, NO production and *in vitro* angiogenesis-tube formation (Su et al., 2014b). The role of TRPA1 was further confirmed using matrigel plugs *in vivo* in TRPA1 knockout mice, whereby simvastatin—induced angiogenesis was partially reduced (Su et al., 2014b).

## Conclusion

ECs activity, such as proliferation, migration, and survival is required for angiogenesis under both physiological conditions, (vessel growth and renewal) and pathological conditions, (cardiovascular diseases and tumors initiation and progression). Alteration of these functions resulted from exaggerated or reduced bioavailability of various downstream effectors of VEGF receptors. For example increased Akt and ERK activation following sustained VEGFRs-VEGF interaction induces tumor angiogenesis and growth, whereas, reduced Nitric oxide (NO) production seemed to cause endothelial dysfunction such as deficiency in vascular relaxation. It is now evident that TRP channels are critically involved in physiological and pathological angiogenic process. By controlling Ca^2+^ homeostasis, different TRP isoforms are activated by pro-angiogenic stimuli that evoke ECs proliferation and migration, as well as the formation of new capillary derived either from ECs or from EPCs. Nevertheless, considerable work is needed to fully understand why many TRPs from different subfamilies are activated by similar pro-angiogenic stimuli such as VEGFs, and whether these TRPs might associate between them to promote their angiogenic effect. To the best of our knowledge, and from the point of view of angiogenesis, the organization and interactions between closely related TRP channels have not been addressed. Several questions still remain unsolved concerning the role or TRP channels in angiogenesis such as are different TRPs located in microdomains with different VEGF-receptors? Are different Ca^2+^ signals generated by these TRP complexes inducing different cellular functions? Further studies will definitely clarify these and other functional aspects.

In light of the reported findings, the search of selective pharmacological blockers or activator of TRP channels stands out among the strategies for obtaining promising molecular drugs to normalize angiogenesis or for anti-angiogenic therapies to prevent tumor neovascularization.

## Author contributions

TS, A-MK, and JR conceived the concept of the review. TS, A-MK, LG, GS, and JR wrote the review. SR, GW, and A-MK designed and formatted the figures. TS, LG, SR, GW, GS, A-MK, and JR read and edited the review manuscript.

### Conflict of interest statement

The authors declare that the research was conducted in the absence of any commercial or financial relationships that could be construed as a potential conflict of interest.
